# Family Structure Transitions: Prevalence and Physical Health Effects in Ethiopia, India, Peru, and Vietnam

**DOI:** 10.1007/s10826-021-02148-2

**Published:** 2021-11-03

**Authors:** Rebecca Oldroyd, Shazia Rahman, Laurie F. DeRose, Kristin Hadfield

**Affiliations:** 1 Queen Mary University of London, Mile End Road, Bethnal Green, London, E1 4NS UK; 2 The Catholic University of America, 620 Michigan Avenue, Washington, DC 20064 USA; 3 The Maryland Population Research Center, Morrill Hall, College Park, MD 20742 USA; 4 School of Psychology, Trinity College Dublin, College Green, Dublin 2, D02 PN40 Ireland; 5 Trinity Centre for Global Health, Trinity College Dublin, College Green, Dublin 2, D02 PN40 Ireland

**Keywords:** Family structure transitions, Low-and middle-income countries, Cross-cultural, Family relationships, Health

## Abstract

This study aimed to identify the prevalence and physical health consequences of family structure transitions among children in Ethiopia, India, Peru, and Vietnam. In many high-income countries, family structure transitions are common, and research suggests that they can lead to worse physical health for children. However, we know little about either the prevalence or consequences of family structure transitions for children in low-and middle-income countries, who make up the vast majority of the world’s children. First, we estimated the number of family structure transitions by age 12 using four rounds of Young Lives data from four low-and middle-income countries (*N* = 8062, Ethiopia, India, Peru, and Vietnam) and validated our prevalence estimates with another dataset from these same countries. The proportion of children experiencing a family structure transition by age 12 was: 14.8% in Ethiopia, 5.6% in India, 22.0% in Peru, and 7.7% in Vietnam. We put these estimates in context by comparing them to 17 high- and upper-middle-income countries. Second, using linear mixed models, we found that family structure transitions were not directly associated with worse physical health for children in Ethiopia, India, Peru, and Vietnam. Children in Peru experienced higher rates of family structure transitions relative to children in the other Young Lives countries, and similar rates to many of the 17 comparison countries, yet physical health was unaffected. It is possible that in low-and middle-income countries, the environment may overwhelm family stability as a determinant of physical health.

## Highlights

- The prevalence of children experiencing a family structure transition by age 12 in Ethiopia, India, Peru, and Vietnam was 5.6–22.0%: a wide-range, similar to what has been observed in high-income contexts.
- Family structure transitions are not directly associated with children’s general physical health in Ethiopia, India, Peru, or Vietnam.
- Family structure transitions do not influence children’s general physical health trajectories over time in Ethiopia, India, Peru, or Vietnam.

Family structure transitions are changes in household composition caused by a change in the parents’ romantic relationships (e.g., moving from a two-biological-parent family to a single-parent family following a divorce or separation, Cavanagh & Fomby, 2019; Hadfield, Ungar, et al., 2018). This can be measured by determining whether the parents’ cohabiting relationship status changes over time. While some of the family instability literature includes non-parental family transitions (e.g., sibling or grandparent transitions, Mollborn et al., 2012; Perkins, 2017, 2019; Sun & Li, 2014), this paper—much like the majority of the family instability literature - focuses exclusively on parental transitions. Although it is widely acknowledged that marriage and parenting cultures vary tremendously around the world, we do not have evidence of how much the prevalence of transitions varies across the Global South, nor how it compares to the Global North.

This paper estimates the proportion of children experiencing a family structure transition by age 12 in four low- and middle-income countries (LMICs) (Ethiopia, India, Peru, and Vietnam). Given the broad literature on the potential consequences of family structure transitions for children’s health and wellbeing, we also test whether family structure transitions lead to worse physical health for children in these four countries. In doing this, we integrate the two types of family demography highlighted by Smock and Schwartz (2020): (1) identifying trends, and (2) exploring the social and cultural factors associated with family structure transitions.

The majority of the family structure transition research focuses on high-income countries (See Table 1 for country income definitions), particularly the United States (Hadfield, Amos, et al., 2018). In many high-income countries, the familial landscape has changed dramatically in recent decades: 56% of children in the United States and 32% of children in the United Kingdom born to married parents now experience at least one family structure transition by age 12 (Brown et al., 2016; DeRose, Lyons-Amos, et al., 2017; Santesteban-Echarri et al., 2016; Smock & Schwartz, 2020). By contrast, children in Belgium, Spain, and Poland born to married parents are much less likely to experience a family structure transition by age 12 (8%, 5%, and 6%, respectively) (DeRose, Lyons-Amos, et al., 2017).

**Table 1:** Definitions of high, upper-middle, lower-middle, and low-income countries

Country income status	Definition
High income	Gross national income per capita of $12,696 or more
Upper-middle income	Gross national income per capita between $4096 and $12,695
Lower-middle income	Gross national income per capita between $1046 and $4095
Low income	Gross national income per capita of $1045 or less

Definitions are provided by The World Bank (2021) and reflect the 2022 fiscal year

So, we know that the prevalence of family structure transitions varies greatly between high-income countries, but what about LMICs, where 90% of the world’s children live (Blum & Boyden, 2018)? We know little about the prevalence of family structure transitions for these children. DeRose and colleagues’ (2017) work on family instability across the globe provides a rare comparison of the proportion of children experiencing a family structure transition by age 12 in 17 countries. However, all of these were high- (13 countries) or upper-middle-income (4 countries); there is an unfortunate lack of information on families living in LMIC contexts. The consequences of family structure transitions in LMICs have also received disproportionately little attention relative to high-income contexts, even though some of the mechanisms through which transitions affect children are likely to be pertinent globally. The instability hypothesis suggests that family structure transitions cause stress which, in turn, leads to a myriad of negative child outcomes (Fomby & Cherlin, 2007; Wu & Martinson, 1993).

Research conducted in high-income countries suggests that transitions can lead to number of negative outcomes for children, including an increased risk of obesity, asthma, and worse general physical health (Augustine & Kimbro, 2015; Bzostek & Beck, 2011; Wickrama et al., 2013). However, the literature on the impacts of family structure transitions is mixed: transitions do not always lead to stress or to worsened outcomes for children (Hadfield, Amos, et al., 2018), which suggests that there may be circumstances under which transitions have neutral or even positive effects (Brown, 2006). If a family structure transition involves the entrance of a caregiver, for example, this could reduce the stress and the negative outcomes associated with transitions because of the financial, instrumental, and social support the additional caregiver could offer. If a family structure transition involves the exit of a caregiver, though, this will likely lead to a reduction in resources (e.g., money, time) which could increase the stress and the negative outcomes associated with experiencing a transition. That is, although the instability hypothesis suggests that family structure transitions will lead to negative outcomes, evidence for this is inconsistent.

The studies that have explored how family structure impacts child outcomes in LMICs show that living in non-married family structures (i.e., single-parent, divorced, or cohabiting families) can increase children’s risk of mortality, anaemia, and living in poverty (Bhuiya & Chowdry, 1997; Clark & Hamplova, 2013; Cuesta, et al., 2017; DeRose, Salazar-Arango, et al., 2017; Schmeer, 2013). These studies unfortunately tell us little about how children’s outcomes change *over time*, because they focus on between-person rather than within-person differences. For example, they compare the outcomes of children born to married versus divorced mothers. Examining the impacts of children’s static family structures at one timepoint does not reflect the reality that many children experience changes in their family structure during childhood. As per the instability hypothesis, these changes – rather than being in one family structure or another at a given point in time – may be what is harmful to child outcomes (Fomby & Cherlin, 2007; Wu & Martinson, 1993). That is, it is hypothesized that family structure *transitions* matter more than family structure per se for children’s outcomes because transitions lead to stress, and this stress leads to negative outcomes for children.

We used the younger cohort data from the Young Lives study, which follows children from age 1 to 15 in Ethiopia, India, Peru, and Vietnam (Boyden, 2018). LMIC contexts are crucial to study because we know that many young people from LMICs have poor physical health due to a range of environmental and socioeconomic factors (Boyden, 2018). The physical health of those living in impoverished areas is particularly at risk given the association between poverty and poor physical health (Dornan & Woodhead, 2015; Marmot, 2005; Nikulina, 2014). Living in poverty also affects people’s access to healthcare; in all four of the Young Lives countries—although there are governmental strategies aimed at improving access to healthcare (Global Health Workforce Alliance, 2021; Habtemariam & Semegn, 2018; Le et al., 2010; The Commonwealth Fund, 2020; World Health Organization, 2021)—those living in rural/poorer areas are less likely to have healthcare access (Young Lives, 2021a). Thus, those who are already at risk of having poor health are also at a greater risk of not being able to access healthcare. Receiving healthcare could also push people further into poverty due to the cost of travelling to healthcare facilities and receiving treatment and medicines. This is true in India, for example, where “health expenditures are responsible for more than half of Indian households falling into poverty” (Balarajan et al., 2011, p. 505). Experiencing a family structure transition—particularly one involving the loss of a caregiver and therefore resources (e.g., money)—could be detrimental to children’s health (Augustine & Kimbro, 2015; Bzostek & Beck, 2011; Wickrama et al., 2013) because it could exacerbate existing disadvantage and poverty which could, in turn, impair their physical health (Marmot, 2005). It is important to determine whether family structure transitions impact health among young people in LMICs so that interventions and policy can be implemented to support these families. Alternatively, living in a context with high levels of poverty may mean that transitions have a limited impact on children’s physical health, because other challenges such as access to clean water, malnutrition, and child labour may be more impactful to children’s physical health than a family structure transition.

This paper has two aims. The first is to identify the prevalence of family structure transitions in Ethiopia, India, Peru, and Vietnam. To this end, we used four rounds of Young Lives data on children from age 1 to 12 to identify the number and types of family structure transitions children experienced by age 12. We focused on age 1 to 12 in order to be able to compare the Young Lives prevalence estimates with well-established estimates of the prevalence these transitions by age 12 in high-and upper-middle-income countries (Brown et al., 2016; DeRose, Lyons-Amos, et al., 2017). The second aim is to identify the effects of family structure transitions on children’s physical health between age 1 and 15. We used linear mixed models to examine within- and between-person changes in physical health over time, as a function of family structure transitions. We control for individual fixed effects, allowing us to account for the selection hypothesis (Wu & Martinson, 1993) which states that some parents possess characteristics which make them more likely to experience multiple family structure transitions, and their children more likely to experience negative developmental outcomes. We ran additional models to examine the influence of family structure transitions on children’s physical health trajectories. In line with some existing research conducted in high-income countries (e.g., Bzostek & Beck, 2011), we hypothesized that children who had experienced a family structure transition would have worse physical health than those who had not. We also hypothesized that the impact of family structure transitions would be worse in countries with a lower prevalence of transitions, because they are less normative, and thus potentially more stressful (Ryan & Claessens, 2013).

## Methods

### Data

We used data from the Young Lives study (*N* = 8062 study children and their primary caregivers; 52% male at baseline), which is a study coordinated by the University of Oxford that aims to examine childhood poverty in Ethiopia, India, Peru, and Vietnam (Boyden, 2018). The study obtained ethical approval before each pilot and round of data collection, and informed consent was provided by everyone involved in the study (i.e., study children, caregivers, and community members, see https://www.younglives.org.uk/content/research-ethics for more information). The study’s goal is to understand the causes and consequences of poverty, and how poverty is transmitted across generations, in order to inform policy and shape poverty reduction strategies (Young Lives, 2017). In line with the aims of the study, the Young Lives study over-samples poorer areas; they used purposive and semi-purposive sampling to include 20 clusters which cover a wide range of demographic regions to reflect a variety of children’s experiences in each country (Young Lives, 2018). Although the Young Lives study is not nationally representative, it has been compared with other nationally representative datasets sampling children from the same countries (e.g., the Demographic and Health Survey), and this comparison revealed that the Young Lives sample adequately reflects the diversity of children and families in each country (Young Lives, 2021b). For more information about the sampling and recruitment process in the Young Lives study, see https://www.younglives.org.uk/content/our-research-methods. Data collection began in 2002, and currently spans 20 years, with six rounds of data available. The sixth round of data was collected during the Covid-19 pandemic and was not included in our analyses. The study consists of four surveys (household, child, school, and community), alongside qualitative methods. We used the household and child surveys to determine household composition and children’s physical health. The primary caregiver completed the household survey at all rounds, and the child completed the child survey from age 8 onwards.

When identifying the prevalence of family structure transitions, we used rounds one to four (ages 1 to 12). This was so that we could compare the number of children experiencing family structure transitions by age 12 with established prevalence statistics from Brown et al. (2016) and DeRose, Lyons-Amos et al. (2017) for 17 countries. These statistics from high- and upper-middle-income countries focus exclusively on children born to married mothers, so it is likely that they produced conservative estimates of transitions, because being born into a single-parent or cohabiting family seems to increase the likelihood of experiencing a family structure transition (Ryan & Claessens, 2013). For our second aim of examining the effects of family structure transitions on children’s physical health, we used rounds one to five (ages 1 to 15) of the Young Lives data in order to include the maximum amount of data possible.

Because of the large gaps between rounds in the Young Lives data—which opens up the possibility of undercounting family structure transitions—we wanted to validate our prevalence estimates using another dataset. To do so, we compared our prevalence estimates with estimates obtained using monthly union history calendar data from the Demographic and Health Surveys (DHS). The DHS data comes from: the 2005 Ethiopia DHS, the 2005-06 India DHS, the 2012 Peru DHS, and the 2002 Vietnam DHS. The Vietnam data are a nationally representative sample of ever-married women aged 15 to 49; in the other three countries the data are nationally representative samples of *all* women aged 15 to 49. The monthly union history calendars date back approximately six years (72 months) from the interview date, and therefore did not allow us to measure transitions in the first 12 years of life for the same children. We measured transitions in the first six years of life using children less than 1 year old at the beginning of the union history data (age 6 at interview), and transitions from age 6 to 12 using children age 12 at interview. Combining these measures into a single estimate for the first 12 years of life is tantamount to assuming that the probability of later transitions is independent of earlier ones (clearly false), but provides the best possible estimate from the six-year monthly union histories. DHS union history calendars are subject to recall errors as they ask women to report on unions from six years before the interview. In contrast, household structure was measured contemporaneously in each round of the Young Lives data. However, the DHS data are monthly, and therefore do not undercount transitions by missing multiple transitions between distantly spaced rounds in the way that the Young Lives data might. Although family structure transitions are measured differently in the DHS data than in the Young Lives data, using the DHS data allowed us to validate our Young Lives prevalence estimates, and address some limitations of the Young Lives data.

The Young Lives sample included 8062 study children aged 1 year old in 2002. When looking at the prevalence of family structure transitions, we excluded children who left the household at any of the four rounds; note though that this is only a very small group of children (Ethiopia: *N* = 21, India: *N* = 67, Peru: *N* = 0, and Vietnam: *N* = 19). We also excluded children who experienced the death of a parent(s), because this is a conceptually different type of transition compared to the types of transitions we were interested in (Ethiopia: *N* = 177, India: *N* = 116, Peru: *N* = 46, and Vietnam: *N* = 64). We further excluded children who only had one round of household data because we could not determine whether they had experienced a family structure transition (Ethiopia: *N* = 87, India: *N* = 62, Peru: *N* = 62, and Vietnam: *N* = 20). Finally, we excluded children who only had two non-consecutive rounds of household data, because the gaps between these rounds were so large that we could not accurately estimate the prevalence of transitions for these children (Ethiopia: *N* = 4, India: *N* = 0, Peru: *N* = 14, and Vietnam: *N* = 2). After excluding these participants, the final sample used for estimating the prevalence of family structure transitions was *N* = 7310 (*N* = 1715 in Ethiopia, *N* = 1769 in India, *N* = 1930 in Peru, and *N* = 1896 in Vietnam).

When looking at the effects of family structure transitions on children’s physical health, we used the same exclusion criteria as above, but the number of children who fit each exclusion criteria is different because we included a fifth round of data. The number of children who left the household at any of the five rounds was still very small (Ethiopia: *N* = 49, India: *N* = 68, Peru: *N* = 3, and Vietnam: *N* = 31). The number of children who experienced the death of a parent was slightly larger than the previous analytic sample (Ethiopia: *N* = 211, India: *N* = 166, Peru: *N* = 71, and Vietnam: *N* = 84). The number of children who only had one round of household data was: Ethiopia: *N* = 87, India: *N* = 61, Peru: *N* = 60, and Vietnam: *N* = 18. Finally, the number of children who only had two non-consecutive rounds of household data was: Ethiopia: *N* = 4, India: *N* = 0, Peru: *N* = 12, and Vietnam: *N* = 2. After excluding these participants, the final sample used to examine the effects of family structure transitions on children’s physical health was *N* = 7143 (*N* = 1650 in Ethiopia, *N* = 1728 in India, *N* = 1895 in Peru, and *N* = 1870 in Vietnam).

### Measures

#### Family structure

We classified children’s family structures based on where the biological mother, biological father, and stepparents (if any) were living (in the same household as the Young Lives child or not), together with information about who acts as the child’s primary caregiver. We did this using the household roster, which listed the number of people in the household and their relationship to the Young Lives child. In total, there were seven possible family structure categories. Three of these described children in parental care: (1) both biological parents in the household, (2) one biological parent in the household and no romantic partner in the household (regardless of whether the mother/father had a partner living elsewhere), and (3) stepfamilies in which the biological parent and their romantic partner lived in the household. Children not in parental care were classified into grandparent-headed, sibling-headed, aunt/uncle-headed, and “other” families.

#### Family structure transitions

In line with the existing family structure transition literature (e.g., Brown, 2006; Cavanagh & Fomby, 2019; Hadfield, Ungar, et al., 2018), we classified a family structure transition as a change in the romantic relationship status of parents residing in the household. Therefore, we only counted a change as a family structure transition if it involved a move between a two-biological-parent family, single-parent family, or stepfamily. The vast majority of the Young Lives children in all four countries lived in these family structures (Table 2). Transitions to or from grandparent-headed, sibling-headed, aunt/uncle-headed, or “other” households were not included in the calculation of family structure transitions to be consistent with our definition of family structure transitions and other work in this area. A score of 1 was given where the child experienced a transition and a score of 0 if their family structure remained the same from one wave to the next.

**Table 2:** Percentage of children living in each family structure at age 1 (round one) and age 12 (round four) in Ethiopia, India, Peru, and Vietnam

	Age 1 (round one)				Age 12 (round four)			
	Ethiopia	India	Peru	Vietnam	Ethiopia	India	Peru	Vietnam
Two-biological-parent family	84.0	99.0	85.1	96.6	68.4	86.3	69.2	89.1
Single-parent family	12.7	0.5	13.5	2.4	18.7	8.6	18.4	5.9
Stepfamily	0.5	0.0	0.4	0.6	3.5	1.2	6.9	1.1
Grandparent-headed household	2.1	0.2	0.6	0.4	6.9	2.6	4.5	3.3
Sibling-headed household	0.1	0.0	0.0	0.0	0.6	0.2	0.4	0.1
Aunt/uncle-headed household	0.3	0.0	0.1	0.0	1.9	1.2	0.6	0.5

Six of the possible seven household structures are described here, because the “other” option was only available at round one. Analytic sample in each country: *N* = 1715 in Ethiopia, *N* = 1769 in India, *N* = 1930 in Peru, and *N* = 1896 in Vietnam

#### Physical health

The two items that measured children’s general physical health in the data were: “Compared to other children, would you say [child’s] health is the same, better, or worse?”, and “In general, would you say [child’s] health is very poor, poor, average, good, or very good?”. The availability of these items differed depending on the country and the round of data collection (Oldroyd, 2020). To allow us to make direct comparisons between countries, we used variables that were available at the same rounds across all four countries; we used the “Compared to other children…” variable at rounds one and two, the “In general…” variable at rounds three and four, and both of these variables at round five. We chose to combine the variables in round five because they were moderately to strongly correlated in all four countries at round five (*r* = 0.3 to 0.5, *p* < 0.05). There was a significant, positive correlation between the adjacent general physical health items at all rounds, in all four countries (Oldroyd, 2020). Given the large gaps between each round of data collection in the Young Lives study (approximately three-to-four years), it makes sense to focus on children’s general physical health because this construct is measured using at least one of the same two general physical health items from age 1 to 15, as opposed to other outcome measures which change depending on the developmental stage of the child (i.e., cognitive measures). Higher scores indicate better general physical health.

#### Shocks

The “shocks” scale is a checklist of events that could have affected the household. In total, there were 44 items. We used the eight items from the checklist that measured environmental events, such as “too much rain or flooding” and “pests or diseases affecting livestock”, because we wanted to control for some external environmental influences on children’s physical health (Kousky, 2016). We used the same items in every round in all four countries. Higher scores indicate more exposure to environmental shocks.

#### Premature birth

In round 1, the mothers were asked “was the child born before expected?”. If they answered “yes” (1), that indicated that the child was born prematurely (no = 0). This item was the same in all four countries.

#### Child sex

This was a binary variable with female coded as 0 and male coded as 1. This variable was the same in all four countries.

### Analytic Strategy

We conducted a frequency analysis to determine the proportion of Young Lives children living in each family structure at each round. We used logic statements to identify whether children had moved between two-biological-parent, single-parent, and stepfamilies at each round. We then identified the percentage of children experiencing a move between these three family structures (i.e., a family structure transition) at each round, as well as the total number of transitions each child experienced across the four rounds.

To address our second aim, we used linear mixed models (LMMs; SPSS Version 25) to assess the association between family structure transitions and children’s physical health. This method is appropriate because of the nested data structure: time (Level 1) is nested within participants (Level 2). This method accounts for selection effects (i.e., parental characteristics that select families both into more transitions, and into worse child outcomes, Wu & Martinson, 1993) by looking at within-person as well as between-person change. That is, LMMs compare children to *themselves* over time. These models do not assume independence of data, which is appropriate for longitudinal analyses in which individual’s scores are correlated over time. LMMs can also handle missing or unevenly spaced data (Shek & Ma, 2011), which is ideal for longitudinal data that is prone to attrition. That said, one of the major strengths of the Young Lives data is that the attrition rates “are the lowest ever reported in the longitudinal studies literature” (Outes-Leon & Dercon, 2008, p. 8). From rounds one to five (ages 1 to 15), attrition rates were: 4.5% in Ethiopia, 3.0% in India, 8.2% in Peru, and 2.3% in Vietnam. Children who dropped out of the study at any time between rounds one and five were more likely to be impoverished and born to single-parent families, compared to children who remained in the study at all five rounds (*p*s < 0.05), which is typical in cohort studies. However, LMMs do not use listwise deletion and so these participants are still included in the analytic sample and contribute to the models, they just have fewer rounds of data than the other participants.

The predictors in the analysis testing our second aim were family structure transitions (no = 0, yes = 1), child sex (female = 0, male = 1), premature birth (no = 0, yes = 1), and shocks (number of shocks). The predictor of interest was family structure transitions; child sex, premature birth, and shocks were control variables. The outcome variable was children’s general physical health. As a sensitivity analysis, we ran the models both including and excluding children who had experienced the death of a parent(s). The number of children who experienced the death of a parent(s) in our analytic sample was: Ethiopia: *N* = 211, India: *N* = 156, Peru: *N* = 82, and Vietnam: *N* = 80. Including these participants did not affect the pattern of results, so we excluded them from the analysis to be consistent with our definition of a family structure transition (Cavanagh & Fomby, 2019; Hadfield, Ungar, & Nixon, 2018). We ran the models with the intercept centered at round one (age 1) and round five (age 15) (Singer & Willett, 2003). We tested this because many children only experienced a family structure transition at later rounds, and therefore differences in general physical health resulting from these transitions may only be apparent towards the end of the study (Singer & Willett, 2003). This did not affect the pattern of results, so we present the findings with the intercept centered at round five (age 15), representing children’s general physical health status when they were 15 years old.

A series of LMMs using maximum likelihood estimation were fit to the data, in which physical health over time was modeled as a linear function of family structure transitions from age 1 through 15. Intercepts (physical health at age 15) and slopes (trajectories over time) were allowed to vary by participant. First, we ran unconditional mean models to examine individual variation in physical health without accounting for time. We then ran unconditional linear growth models to examine individual variation in physical health over time. Finally, we ran the model including the time-variant predictors (family structure transitions and shocks), and the time-invariant predictors (sex and premature birth).

Further, we ran a series of additional LMMs to determine whether experiencing a family structure transition influenced children’s physical health trajectories (i.e., slope, or rate of change). Where the analyses described above treated family structure transitions as time-varying, here we made them fixed effects and included a time * transition variable to examine the impacts of family structure transitions on the *slope* of children’s physical health. We ran three models for each country: first with a transition variable which reflected whether the child had experienced at least one family structure transition between the age of 1 and 15 (0 = no transition, 1 = at least one transition), then with a transition variable reflecting whether the child had experienced a family structure transition between the age of 1 and 5, and finally with a transition variable reflecting whether the child had experienced a family structure transition between the age of 5 and 8. We could not run this analysis with a transition variable that reflected experiencing a family structure transition at between age 8 and 12, or 12 and 15, because a minimum of three rounds of outcome data are required for this analysis (Curran et al., 2010). The aim of this analysis was to see whether experiencing a family structure transition put the child on a different physical health trajectory than if they had not experienced a transition, and to examine the effect of experiencing a family structure transition at different ages.

Using Soper’s (2016/2017) calculator, a post-hoc power analysis was conducted for the analyses in each of the four countries. Sample size for the within-person effects was *N* multiplied by the number of measurement occasions (five) minus one. Sample size for the between-person effects was the total sample size in each country. Power was calculated accounting for the three control variables (sex, premature birth, shocks), and the two predictor variables (family structure transition, time). In all four countries, within-person power was 100% to detect a small effect (0.02), and between-person power was 99% to detect a small effect (0.02). This suggests that we had sufficient statistical power in the present sample to detect small between- and within-person effects of family structure transitions on children’s physical health. To correct for familywise error, a Bonferroni correction was applied. The standard *p* value (0.05) was divided by the number of analyses run in each country (4) to generate a new *p*-threshold of 0.0125.

## Results

### Family Structure

First, we identified the family structures that children were living in at each round from ages 1 to 12 in Ethiopia, India, Peru, and Vietnam. The most common type of family structure in all four of the countries and at every round was the two-biological-parent family. Single-parent families were the second most common family type in all countries, followed by stepfamilies. Grandparent-headed, sibling-headed, and aunt/uncle-headed households accounted for 9.4% of family structures in Ethiopia, 4.0% in India, 5.5% in Peru, and 3.9% in Vietnam at age 12 (Table 2).

The average household size at round one—when the child was 1 year old—was: 5.72 in Ethiopia, 5.42 in India, 5.70 in Peru, and 4.90 in Vietnam. The proportion of children living in multigenerational households (i.e., households that included at least one grandparent) at round one was: 10.8% in Ethiopia, 31.7% in India, 29.1% in Peru, and 34.8% in Vietnam. The proportion of children living with extended family members other than grandparents (i.e., at least one aunt or uncle) was: 13.7% in Ethiopia, 30.3% in India, 27.1% in Peru, and 20.8% in Vietnam. Living in a multigenerational or extended kin household is not mutually exclusive with living in a two-biological-parent family, single-parent family, or stepfamily. That is, a child could live with their two biological parents but also have a grandparent in the household, and therefore be living in both a two-biological-parent household and a multigenerational household.

### Family Structure Transitions

**Fig. 1:**
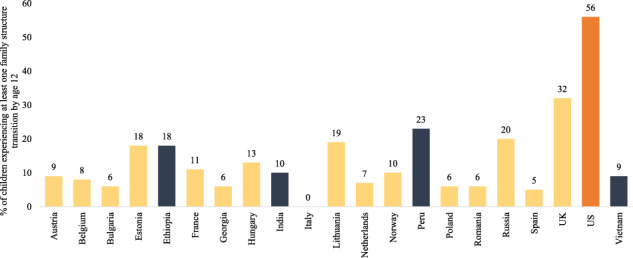
Percentage of children who experienced at least one family structure transition from age 1 to 12 in Ethiopia, India, Peru, and Vietnam, compared with rates in 17 high- and middle-income countries. The bars in blue are our prevalence estimates using the Young Lives data. The bar in orange is from Brown et al. (2016), and the bars in yellow are from DeRose, Lyons-Amos et al. (2017). The bars in orange and yellow represent children born to married mothers only, so it is likely that these are conservative estimates of family structure transitions by age 12. Unlike the results in the rest of this paper, the data in this figure includes transitions as a result of parental death to allow for simple comparison with Brown et al. (2016) and DeRose, Lyons-Amos et al. (2017) estimates, which include parental death as a family structure transition. The number of children who experienced parental death by age 12 was: Ethiopia: *n* = 177, India: *n* = 116, Peru: *n* = 46, and Vietnam: *n* = 64. Percentages listed above the bars are rounded to the nearest integer, with bars representing exact figures

Second, we counted the prevalence of family structure transitions in Ethiopia, India, Peru, and Vietnam. A family structure transition involved any change between a two-biological-parent family, a single-parent family, and a stepfamily. In the Young Lives data, the proportion of children experiencing at least one family structure transition was highest in Peru (22.0%), followed by Ethiopia (14.8%), Vietnam (7.7%), and India (5.6%) (Fig. 1). This corresponds closely with estimates using the DHS data: Peru (25.4%), Ethiopia (16.4%), Vietnam (6.2%), and India (5.7%). Neither estimate is perfect: The Young Lives data misses transitions during the 3 to 4 years between each round (underestimating transitions), while the six-year time period covered by the DHS data requires us to assume independence of earlier and later transitions (thus overestimating transitions, because children with stable family lives before age 6 are less likely to experience a transition from age 6 to 12, relative to their counterparts). We are encouraged that both data sources suggest similar levels of transitions, and we favour the Young Lives estimates for two reasons. First, undercounting transitions between rounds could greatly affect the count of total transitions, but it would have a much smaller effect on the percentage experiencing at least one transition. Second, the difference between the Young Lives and the DHS estimates are minimal in India and Vietnam where transitions are infrequent, while the DHS estimates are higher than the Young Lives estimates in Ethiopia and Peru where transitions happen more often. The assumption that earlier (by age 6) and later (from age 6–12) transitions are independent would be expected to inflate estimates more where transitions are more frequent overall, which is exactly what we see.

Most children experiencing a family structure transition by age 12 had only one transition (Table 3). In all four countries, the most common type of family structure transition was the transition from a two-biological-parent family to a single-parent family. This most often occurred between the ages of 1 and 5 in Ethiopia, 5 and 8 in Vietnam, and 8 and 12 in India and Peru.

**Table 3:** Percentage of children experiencing a family structure transition(s) by age 12 (round four) in Ethiopia, India, Peru, and Vietnam

	Experienced one transition	Experienced two transitions	Experienced three transitions	Total %
Ethiopia (*N* = 1715)	11.3	3.0	0.4	14.8
India (*N* = 1769)	4.0	1.4	0.3	5.6
Peru (*N* = 1930)	18.2	3.5	0.3	22.0
Vietnam (*N* = 1896)	4.9	2.7	0.1	7.7

When calculating the prevalence of family structure transitions, we used four rounds of data. Therefore, the maximum number of transitions that we could capture in this analysis was three

### Physical Health

Next, we examined the physical health sequelae of family structure transitions among children in all four Young Lives countries from age 1 to 15. By extending to age 15, we saw increases in the number of children experiencing a family structure transition, but the pattern of results remained the same: children in Peru experienced the highest proportion of transitions by age 15 (25.1%), followed by Ethiopia (17.3%), Vietnam (11.0%), and India (7.9%).

There were no statistically significant differences in general physical health for children who experienced a family structure transition relative to those who did not in any of the four Young Lives countries (Table 4). That is, family structure transitions were not directly related to child physical health in Ethiopia, India, Peru, or Vietnam. Further, there were no statistically significant differences in children’s general physical health trajectories for children who experienced a family structure transition relative to those who did not in any of the four Young Lives countries (*p*s > 0.012). Premature birth was not associated with children’s physical health in any of the four Young Lives countries. Boys had slightly better physical health than girls in Ethiopia at the *p* < 0.05 level, but not at our more stringent *p*-threshold of 0.012 (*p* = 0.035). Shocks were significantly associated with children’s physical health in all four of the Young Lives countries at the *p* < 0.05 level and at the more stringent *p*-threshold of *p* < 0.012 in Ethiopia, India, and Peru (*p* = 0.001 in Ethiopia, *p* = 0.002 in India, *p* = 0.004 in Peru, and *p* = 0.013 in Vietnam), with those who experienced more shocks having poorer physical health.

**Table 4:** Parameter estimates for the linear mixed models examining the relationship between family structure transitions and child physical health, controlling for sex, premature birth, and shocks

	Ethiopia (*N* = 1650)				India (*N* = 1728)				Peru (*N* = 1895)				Vietnam (*N* = 1870)			
	*B*	*SE*	*t*	*95% CI*	*B*	*SE*	*t*	*95% CI*	*B*	*SE*	*t*	*95% CI*	*B*	*SE*	*t*	*95% CI*
Intercept	4.05**	0.03	160.00	4.00, 4.10	3.90**	0.04	99.06	3.82, 3.97	3.69**	0.02	194.43	3.65, 3.72	3.27**	0.02	164.06	3.23, 3.31
Family transition	−0.05	0.05	0.95	−0.14, 0.05	−0.02	0.06	−0.30	−0.14, 0.10	−0.00	0.03	−0.10	−0.06, 0.06	−0.06	0.05	−1.33	−0.16, 0.03
Sex	0.06*	0.03	2.12	0.00, 0.11	0.02	0.02	0.77	−0.03, 0.06	0.01	0.02	0.28	−0.03, 0.04	0.05	0.02	1.89	−0.00, 0.09
Premature birth	−0.05	0.05	−1.13	−0.14, 0.04	−0.02	0.04	−0.48	−0.09, 0.06	0.01	0.02	0.36	−0.03, 0.05	−0.01	0.04	−0.16	−0.08, 0.07
Shocks	−0.03**	0.01	−3.27	−0.05, −0.01	−0.04*	0.01	−3.07	−0.07, −0.02	−0.03*	0.01	−2.86	−0.05, −0.01	−0.03*	0.01	−2.48	−0.05, −0.01

The *p*-values for all family transition analyses were >0.05, well above our *p*-threshold of 0.012. -2 Log Likelihood: Ethiopia = 17588.59, India = 17420.31, Peru = 16609.77, and Vietnam = 17825.90

*p < 0.05, **p < 0.001

## Discussion

In this paper, we have measured family structure transitions in contexts in which they are seldom studied. We found that 22.0% of children in Peru experienced at least one family structure transition by age 12, followed by Ethiopia (14.8%), Vietnam (7.7%), and India (5.6%). We also tested whether family structure transitions are an additional challenge compromising the physical health of children from Ethiopia, India, Peru, and Vietnam. Contrary to our hypotheses, we found that family structure transitions had no association with children’s general physical health in any of the studied countries. Family structure transitions also did not alter children’s general physical health trajectories over time in any of the Young Lives countries.

Similar to prevalence estimates in high-and upper-middle-income contexts (DeRose, Lyons-Amos, et al., 2017), we found that the prevalence of family structure transitions within LMIC contexts is wide-ranging (5.6% - 22.0% in the four studied countries). The prevalence of family structure transitions was the lowest in India where individual autonomy tends to be lower, and family and community input regarding partner selection is high (Dommaraju, 2016). Despite generally having autonomy regarding dating and marriage choices (Scroope, 2018a), the prevalence of family structure transitions in Vietnam was relatively low (7.7%). However, although people in Vietnam may have autonomy over who they date, the majority of people think divorce is “never justifiable” (52.2%, Inglehart et al., 2014). This lack of acceptance regarding divorce could be driving down prevalence estimates in Vietnam. We found that a relatively large proportion of children experience family structure transitions in Peru (22.0%), where there has been a decline in the number of married families and an increase in the number of cohabiting and single-parent families (Cuesta et al., 2017). Being born to a cohabiting or single-parent family increases the likelihood of experiencing a transition (Brown et al., 2016; Manning, 2015) which may explain the prevalence of family structure transitions in this country. The prevalence of transitions may not be as high in Ethiopia as it is in Peru because of the stigma regarding single motherhood (Crivello et al., 2019; Newton-Levinson et al., 2014), meaning that a transition from a two-parent family to a single-parent family may be less likely if it will result in a single-mother household.

The transition from a two-biological-parent family to a single-parent family was the most common type of transition in all four countries. However, *when* this transition was most likely to occur differed. In Ethiopia, this transition was most likely to occur between the ages of 1 and 5, whereas it was between 5 and 8 in Vietnam, and 8 and 12 in India and Peru. The differences in the timing of these transitions could have important implications for child development, since transitions during early childhood have been associated with poorer child outcomes than later transitions (Ryan & Claessens, 2013; Pasqualini et al., 2018).

We hypothesised that family structure transitions would be more harmful in countries where they are less normative, but instead we found that transitions were not associated with children’s general physical health in any of the Young Lives countries. It could be that family structure transitions only have a negative impact on children when their lives are relatively stable in other ways. That is, some children from LMICs experience a wide range of social and environmental challenges, such as limited access to sanitised water and malnutrition (Boyden, 2018), which may outweigh any affects that family structure transitions have on children’s physical health. Environmental shocks were associated with the children’s physical health in three of the four countries in these analyses, which suggests these shocks have impacts over and above any potential influence of family structure transitions. Another possibility is that living in multigenerational households or households that include extended kin may act as a protective buffer against the stress and, in turn, the negative consequences associated with family structure transitions, as other family members may be able to compensate for decreased resources as a result of a transition (e.g., instrumental and social support, Mehio-Sibai et al., 2009).

An alternative explanation for our null findings is that family structure transitions could be harmful for some children’s physical health but neutral or even positive for others, and these competing patterns of results could be effectively cancelling each other out. For instance, family structure transitions that involve the entrance of a caregiver (e.g., moving from a single-parent family to a stepfamily) might be beneficial for children’s physical health, because the additional caregiver could provide support (e.g., financial, instrumental, social) which could alleviate the stress and therefore the negative outcomes associated with transitions. Alternatively, family structure transitions that involve the exit of a caregiver (e.g., moving from a two-biological-parent family to a single-parent family) might be harmful to children’s physical health because the loss of support might reduce resources (e.g., money) which might be beneficial for their health. This explanation is not, however, in line with the predictions of the instability hypothesis.

We have used large, longitudinal datasets to explore family structure transitions in understudied contexts, but this approach has also led to some limitations. The Young Lives study collects data every three or four years, and there is no information on family structure during the gaps between each round of data collection. However, we used monthly DHS data to validate our Young Lives prevalence estimates and found that our estimates are consistent with those using the DHS data. We searched for other LMIC datasets (Institute for Fiscal Studies, 2019) to use to either complement the Young Lives data or to add additional LMICs to this analysis, but we were unable to find any that had adequate household variables, spanned from early childhood to age 12, were conducted within the past 20 years, and aligned with the ages of the Young Lives study children.

The physical health variable was another limitation of this study. First, this variable was a survey measure which is less optimal than a direct assessment, because people’s perceptions of what constitutes “poor” or “good” physical health is subjective. Further, the availability of the physical health items in the Young Lives data differed depending on the round of data collection and the country, with some items only being available at one round. Because of the large gaps between each round of data collection, we chose to measure general physical health as this was more likely to be affected by a transition than short-term physical health symptoms, such as “having a fever”. There were two general physical health items, and at least one of them was available at each of the five rounds in all countries (Oldroyd, 2020). Despite only having one out of the possible two general physical health items in rounds one to four, focusing on general physical health made the most theoretical sense, and it was the only facet of physical health that allowed us to address our research question using this data.

Another limitation of our study is that we were unable to capture family structure transitions within stepfamilies. That is, we were not able to identify transitions from living with one stepparent to another stepparent. This is because we only had yes/no information about whether a partner lives in the household, rather than information about who the partner was, and if the partner changed from one round to another. However, one strength of our classification method is that we based it on where the biological parents and their partners were living, which allowed us to categorise stepfamilies that may not personally identify themselves as a stepfamily (Hadfield & Nixon, 2013).

## Conclusion

Our study was the first to count and examine the effects of family structure transitions in the Global South. Our research has highlighted the need for large-scale, longitudinal studies of children living in LMICs that sample children regularly and ask about their family lives. This would enable researchers to better understand these children’s family environments, including a more thorough and accurate estimate of the prevalence and consequences of family structure transitions in these contexts. Overall, there is a need for a more contextualized understanding of how family structure transitions impact children and their families. Our research suggests that family structure transitions are a normative experience for children in at least some countries of the Global South, and that the impact of transitions on these children’s development needs to be understood in context.
